# Sociability strongly affects the behavioural responses of wild guanacos to drones

**DOI:** 10.1038/s41598-021-00234-5

**Published:** 2021-10-22

**Authors:** Natalia M. Schroeder, Antonella Panebianco

**Affiliations:** 1 Red Witral - Instituto Argentino de Investigaciones de Zonas Áridas, CONICET, CC 507, CP 5500 Mendoza, Argentina; 2 Grupo de Investigación en Eco-Fisiología de Fauna Silvestre (INIBIOMA-CONICET-AUSMA-UNCo), Pasaje de la paz 235, CP 8370 San Martín de los Andes, Neuquén Argentina; 3 Facultad de Ciencias Agrarias, Universidad Nacional de Cuyo, Mendoza, Argentina

**Keywords:** Ecology, Zoology, Ecology, Environmental sciences

## Abstract

Drones are being increasingly used in research and recreation but without an adequate assessment of their potential impacts on wildlife. Particularly, the effect of sociability on behavioural responses to drone-associated disturbance remains largely unknown. Using an ungulate with complex social behaviour, we (1) assessed how social aggregation and offspring presence, along with flight plan characteristics, influence the probability of behavioural reaction and the flight distance of wild guanacos (*Lama guanicoe*) to the drone's approach, and (2) estimated reaction thresholds and flight heights that minimise disturbance. Sociability significantly affected behavioural responses. Large groups showed higher reaction probability and greater flight distances than smaller groups and solitary individuals, regardless of the presence of offspring. This suggests greater detection abilities in large groups, but we cannot rule out the influence of other features inherent to each social unit (e.g., territoriality) that might be working simultaneously. Low flight heights increased the probability of reaction, although the effect of drone speed was less clear. Reaction thresholds ranged from 154 m (solitary individuals) to 344 m (mixed groups), revealing that the responsiveness of this guanaco population to the drone is the most dramatic reported so far for a wild species.

## Introduction

The rapid growth of the use and application of unmanned aircraft systems (UAS, or drones) in wildlife research and recreation is unquestionable. The pioneering studies exploring this novel technology appeared in the first decade of the twenty-first century, and focused primarily on evaluating the possibility of detecting different species^1–5^, the feasibility of using UAS in searching for radio-tagged animals^6,7^, and exploring pattern recognition algorithms for detecting animals from drone-derived low-resolution images^8^. In the second decade of the century, publications of studies on the potential uses of drones have multiplied. For just terrestrial drone remote sensing, the number of published articles increased about 28 times between 2011 and 2017, compared with all the entire previous decade (2000–2010), showing a notable exponential growth trend since 2014 (see Fig. 3 in^9^). Similarly, drones are being widely used in recreational activities and are particularly attractive for wildlife sighting. By 2018, there were 184 videos of drones flying over wildlife species in 33 countries on YouTube^10^.

**Figure 1:**
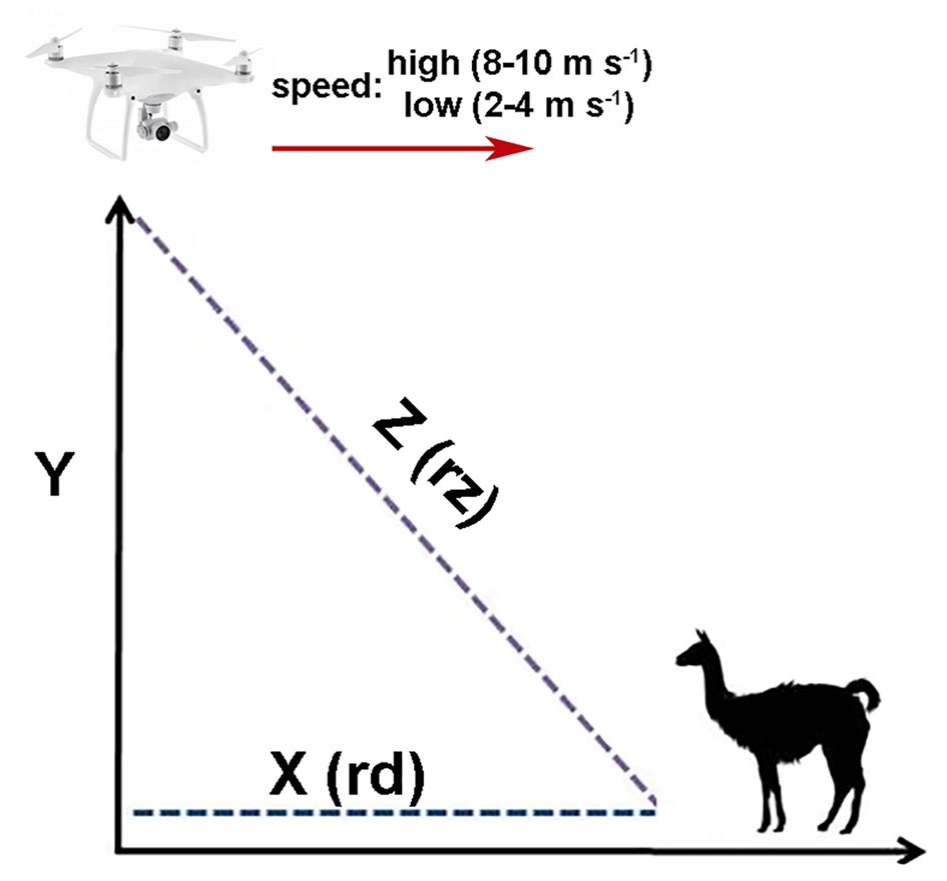
Flight plan for approaching guanacos at La Payunia Reserve. The drone was launched at least 300 m away from the group and flew towards it at constant height and speed. We combined low (2–4 m s^−1^) and high (8–10 m s^−1^) speeds with low and high heights (60 and 180 m AGL, respectively). Flight distance components: Y = flight height; X (rd) = distance between the guanaco group and the drone's projection to the ground, Z (rz) = straight line distance from the guanaco group to the drone. rd = horizontal reaction distance; rz = diagonal reaction distance.

Unfortunately, research on the potential impacts of drones on wildlife for study or sighting has not accompanied this accelerated growth, and only in recent years has it been acknowledged as important to prevent and mitigate drone-associated disturbances. Although internet reports on drone-wildlife interactions have been growing exponentially since 2012 (see Fig. 1 in^11^), by 2018 only 30 articles recording drone effects on wildlife were published, and of these only 50% were actually designed to detect such impacts^10^. The information to date comes mostly from studies on birds and marine mammals, which initially suggested that disturbance was unlikely or imperceptible^12,13^. However, the latest, more experimental studies, which include new species, are showing that the impact of drones can be substantial^11,14^. Flights at higher heights tend to decrease animal responses^11,15–17^, but in order to detect reaction thresholds, more experimental studies at a variety of distances are needed, as well as a deeper understanding of how the life histories of different taxa influence their responses. Notably, the response to drone-induced disturbance of terrestrial mammals is largely unknown.

To assess the potential short-term effect of drones on wildlife, the most widely used approach in the few experimental studies conducted to date has been to record the probability of reaction (i.e., the presence of certain types of behaviour indicating a stimulus is perceived as a disturbance) as a measure of response to drone approach in different contexts, such as flight plans and drone type^12,13,18,19^, environmental conditions and habitat^20^ and across taxonomic groups^16,21^. However, two species may have an equal probability of reacting to the drone advance but differ in the distance to which they react. In this sense, flight distance -the minimum distance at which a wild animal can be approached without fleeing- is more informative than the probability of reaction, as it allows one to (1) obtain a measure of tolerance comparable among individuals, populations or species, (2) evaluate habituation, and (3) estimate reaction thresholds and minimum approach distances that minimise disturbance. Flight distances have been widely used to estimate effects from human disturbance and delineate buffer zones or “safe areas”^22–24^, although, concerning drone-induced disturbance, their use is in its infancy. Flight distances from approaching drones for terrestrial animals, or those staying at ground level, have three components (Fig. 1): horizontal (X = distance between the animal and the drone's projection to the ground), vertical (Y = flight height), and diagonal (Z = straight line distance from the animal to the drone). The three components are mathematically related by Eq. (1)

1 $${Z}^{2}={X}^{2}+{Y}^{2}$$

To our knowledge, only two studies to date have evaluated flight distance as a measure of the animals' response to the drone's approach^11,25^, and only one of these has been on mammals. Penny et al.^25^ used horizontal distance X (Fig. 1) as an independent factor affected by flight height to evaluate the habituation of white rhinoceros (*Ceratotherium simum simum*) and anti-poaching tactics. However, it is likely that animals make a joint evaluation of both the flight height (Y) and the horizontal approach of the drone at that height (X) in a two-dimensional way. Because of this, the Z component (Fig. 1) can be especially informative and useful to detect reaction thresholds and flight heights that minimise the disturbance.

Drone approach (like other human disturbances) may evoke responses analogous to anti-predatory behaviour in wildlife, even when this source is new to the prey’s evolutionary history, because both stimuli pose similar trade-offs between avoiding perceived risk and activities like feeding or parental care^26,27^. In this conceptual framework, sociability has been proposed as a mechanism to improve the effectiveness of such responses. Prey animals living in groups may reduce individual predation risk through two mechanisms that have been widely documented in the literature: (1) collective vigilance among group members, which increases the possibility of detecting predators^28^ and may result in early flight^29^; or (2) dilution effect, which means that grouped prey animals perceive greater per capita safety than solitary individuals^30,31^. As group size increases, flight distances are expected to be greater if there is collective vigilance and shorter if there is a dilution effect. To date, the studies assessing the influence of group size on the behavioural reaction of wildlife to drone flights have lacked an analysis in the context of sociability and behavioural theory. Particularly, for terrestrial mammals, these effects remain poorly understood^16,32^.

Group composition is another factor that may affect the decision to flee. For example, in ungulate followers’ type species^33^, females are more cautious when caring for newborns, which are more susceptible to predation. Thus, groups with offspring often have longer flight distances than groups without offspring. This behaviour is frequent in ungulates^22^ and has been recently observed in white rhino mother-calf pairings in response to drone-induced disturbance^25^.

The guanaco (*Lama guanicoe*) is a wild ungulate with a complex social behaviour, which makes it a good model to evaluate how sociability and group composition modulate responses to drone-associated disturbance. Guanaco forms defined social units: (a) family groups, composed of a territorial reproductive male, several females (2–15 individuals), and their offspring, forming highly cohesive and behaviourally synchronized units; (b) solitary territorial males that defend a territory containing no other individuals, either males or females; (c) bachelor male groups, comprised of non-reproductive and non-territorial males of all age classes (usually juveniles), with group size ranging from a few individuals to more than 50; (d) female groups, including adult females, with or without yearlings and offspring, but without an adult male; and (e) mixed non-territorial groups consisting of males and females of all ages, with variable group size, ranging from 15 to hundreds of animals^34,35^.

In a preliminary study, we described the frequency of guanaco responses to the drone’s approach, identifying that behavioural reactions were more evident in groups larger than 15 individuals, even at high height^36^. Here, we build on our previous work by focusing on the role of social traits on the probability and intensity of behavioural reactions using a theoretical framework from which to make predictions and understand why certain responses occur, thereby improving the scope of predictive models^26^. We focused on reaction distances and used them to estimate reaction thresholds, which are virtually unknown aspects of animal responses to drones, and useful for defining sampling protocols that take animal welfare into account. Specifically, we aimed to first evaluate how social aggregation and offspring presence, together with flight plan characteristics (height and speed) influence both the probability of behavioural reaction and the flight distance of wild guanacos to the drone's approach. Previous studies have shown that adult guanacos benefit from grouping by reducing vigilance effort (the well-known "group-size effect"^37^), but maintaining or even increasing their collective vigilance^38^, which would allow them to increase the probability of detecting predators^29^. Assuming that guanacos use collective vigilance to detect the drone, we expected a greater probability of guanaco reaction and flight distances at larger group sizes and in social groups with a greater number of individuals (bachelors, mixed groups). Additionally, we predicted that groups with offspring would be more responsive to the drone’s approach than groups without offspring. Finally, we estimated reaction thresholds and flight heights that minimise disturbance in different social units, using the Z component of flight distance (Fig. 1).

## Results

We recorded behavioural data of groups belonging to different social units (Table 1) for 90 flights, 47 of them at 60 m above ground level (AGL; n = 24 at 2–4 ms^−1^; n = 23 at 8–10 m s^−1^) and 43 at 180 m AGL (n = 21 at 2–4 m s^−1^; n = 22 at 8–10 m s^−1^). Mean launch distance ± S.D. from target guanaco groups was d0 = 529 ± 120 m. Mean total flight time ± S.D. was 218.49 ± 55.39 and 86.98 ± 25.93 s at 2–4 m s^−1^ and 8–10 m s^−1^, respectively. The average wind speed during the flights was 1.82 ± 1.21 m s^-1^ (range of maximum wind speed recorded, min: 0 m s^-1^; max: 6.73 m s^-1^).

**Table 1:** Summary of the social units in which we conducted the flights, indicating mean group size (± standard deviation), group size range, presence of offspring and number of flights conducted (n = 90). In 8 flights, we could not determine the social unit (undetermined). Height is expressed in metres AGL and speed in m s^-1^.

Social unit	Mean group size ± sd	Group size range (min–max)	Presence of offspring	Number of flights (height/speed)							
				60/2–4		60/8–10		180/2–4		180/8–10	Total
Family groups	7.5 ± 4.2	3–17	yes	4		8		2		6	20
Solitary male	1 ± 0	1–1	no	7		7		7		7	28
Bachelors	13.7 ± 13.0	2–42	no	4		4		-		2	10
Female groups	5.8 ± 5.4	2–15	yes	2		-		2		1	5
Mixed groups	41.9 ± 34.3	17–159	yes	6		3		5		5	19
Undetermined	15.7 ± 19.0	2–51	undetermined	1		1		5		1	8

### Probability of behavioural reaction

**Figure 2:**
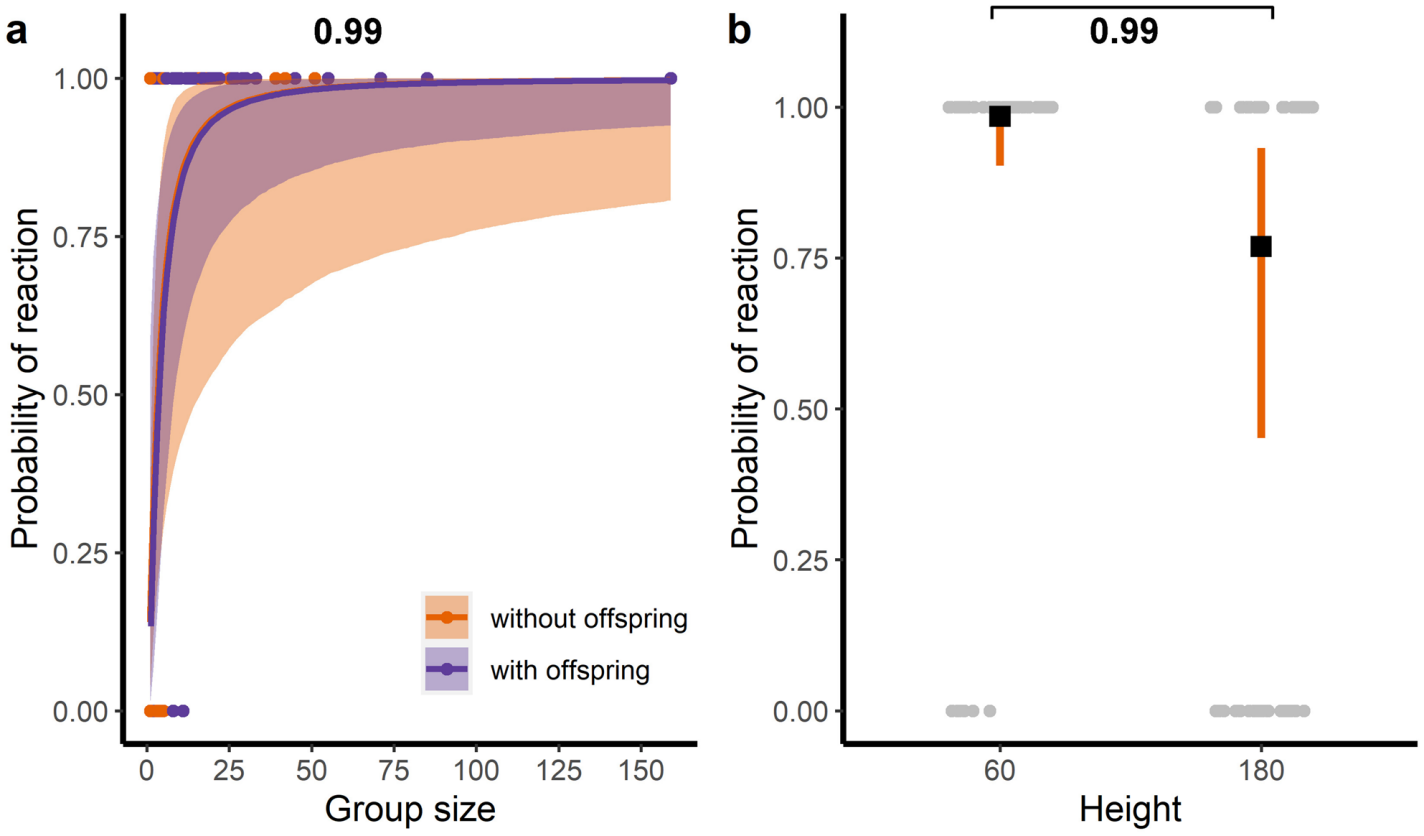
Relationship between the probability of behavioural reaction and (**a**) group size, with and without offspring, and (**b**) flight height, during experimental drone flights. Colour lines and black squares represent mean estimates of the models; colour bands and vertical bars represent the 95% CrI. Dots represent raw data, horizontally jittered in the case of (**b**). Numbers represent the posterior probability of a mean difference between compared estimates. A statistically meaningful effect (presented in bold) can be assumed when the posterior probability of the mean difference between compared estimates is higher than 0.95.

The probability of behavioural reaction of guanacos to the drone’s approach increased with group size. In groups larger than 28 individuals, the probability of reaction reached > 0.90 (Table 2, Fig. 2a). There were no differences in reaction between groups with and without offspring. Regarding flight plan characteristics, only height affected the response variable (Table 2), with an increased probability of reaction of 0.98 (95% CrI 0.90–0.99) at 60 m AGL, compared with a probability of 0.77 (95% CrI 0.45–0.93) 180 m AGL (Table 2, Fig. 2a). We excluded the interaction speed*height in the final model due to poor model fit (i.e., non-acceptable residual patterns). Because all mixed groups reacted, it was not possible to fit a reaction probability model with the type of social unit as an explanatory variable.

**Table 2:** Results from a generalized linear model for the probability of reaction and the horizontal reaction distance (rd). We present estimates of the parameters with their 95% credible intervals (CrI) in brackets. A statistically meaningful effect of a fixed factor (presented in bold) can be assumed if zero is not included within the 95% CrI or if the mean difference between compared estimates is higher than 0.95.

Predictors	Probability of reaction	Horizontal reaction distance (rd)
	Mean estimate (95%CrI)	Mean estimate (95%CrI)
Intercept	0.22 (− 0.93; 1.39)	**5.02 (4.64; 5.40)**
Log(group size)	**1.50 (0.53; 2.46)**	**0.25 (0.12; 0.39)**
Height: 180 m^a^	− **2.92 (**− **4.39; **− **1.44)**	− 0.04 (− 0.60; 0.50)
Speed: 8–10 m s^-1^^b^	0.89 (− 0.43; 2.20)	− **0.36 (**− **0.75; **− **0.04)**
Presence of offspring: yes^c^	− 0.06 (− 1.96; 1.82)	0.01 (− 0.37; 0.37)
Speed: 8–10 m s^-1^ * Height: 180 m	–	0.09 (− 0.62; 0.80)

^a^Reference level: 60 m.

^b^Reference level: 2–4 m s^−1^.

^c^Reference level: without offspring.

### Horizontal flight distance (rd)

**Figure 3:**
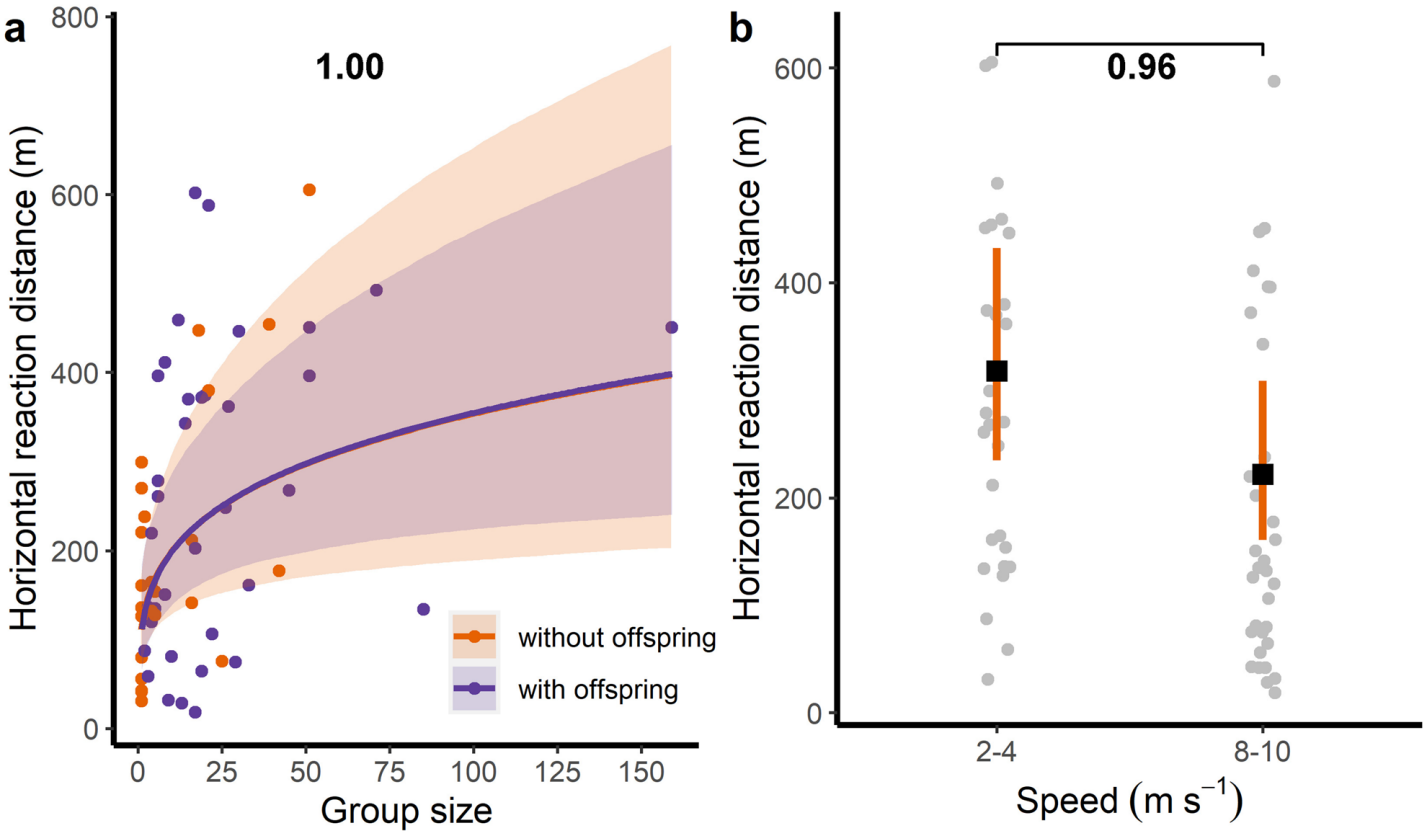
Relationship between the horizontal reaction distance (rd) and (**a**) group size, with and without offspring, and (**b**) flight speed, during experimental drone flights. Colour lines and black squares represent mean estimates of the models; colour bands and vertical bars represent the 95% CrI. Dots represent raw data, horizontally jittered in the case of (**b**). Numbers represent the posterior probability of a mean difference between compared estimates. A statistically meaningful effect (presented in bold) can be assumed when the posterior probability of the mean difference between compared estimates is higher than 0.95.

Mean rd was 232.77 ± 162.48 m. Rd increased with larger group size but not with the presence of offspring in the groups (Table 2, Fig. 3a). In other words, animals in larger groups reacted and fled before those in smaller groups did, irrespective of the presence of offspring. Among flight plan characteristics, only speed had an effect (Table 2, Fig. 3b), with greater rd at 2–4 m s^−1^ (predicted estimate = 313.26 m; 95% CrI 233.67–417.53 m), compared with 8–10 m s^−1^ (predicted estimate = 224.64 m; 95% CrI 162.91–305.21 m). Additionally, compared with solitary males (i.e., reference level; predicted estimate = 104.20 m; 95% CrI 61.59–173.95 m), family groups (predicted estimate = 180.83 m; 95% CrI 113.28–286.13 m) and mixed groups (predicted estimate = 254.14 m; 95% CrI 162.70 -395.80 m) had longer rd, while bachelors’ rd (predicted estimate = 137.98 m; 95% CrI 76.27–249.38 m) and female groups’ rd (predicted estimate = 176.01 m; 95% CrI 71.34–412.41 m) were comparable to the reference level.

### Reaction thresholds and minimum flight heights

**Figure 4:**
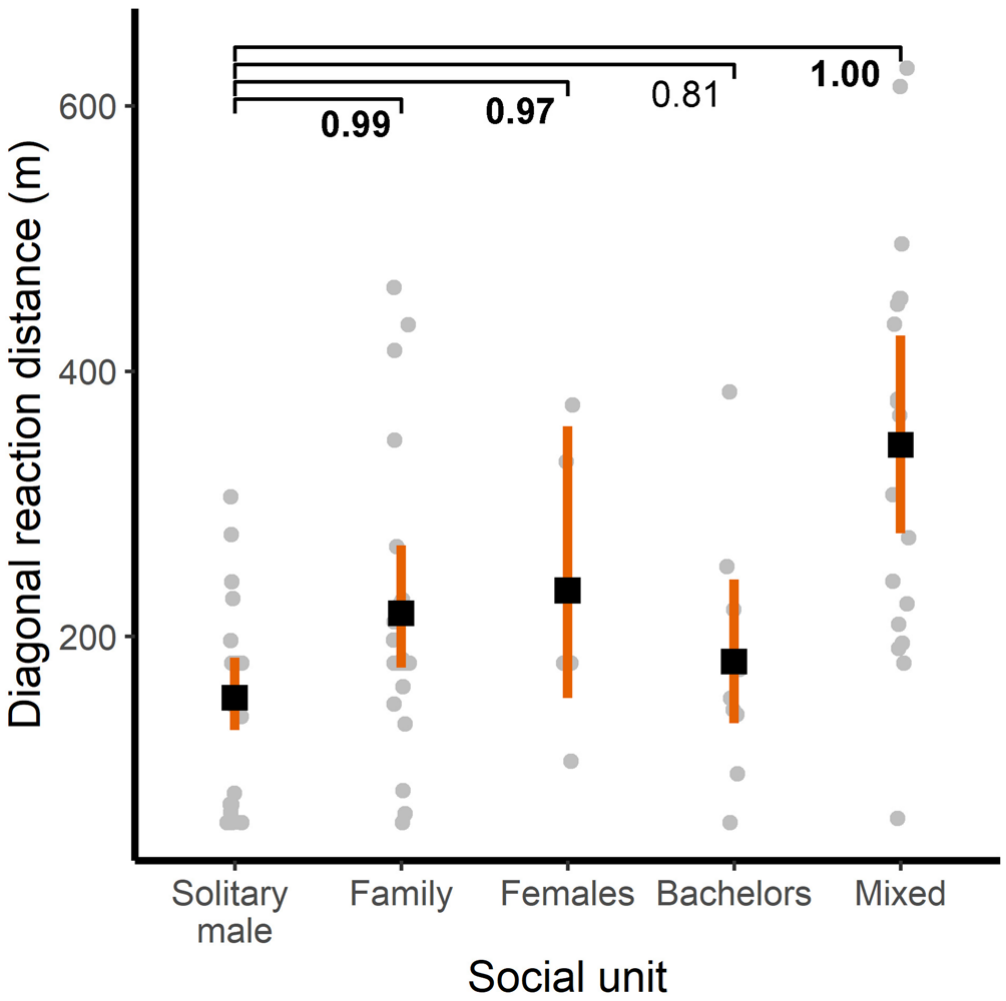
Diagonal reaction distance (rz) and type of social unit during experimental drone flights. Black squares represent predicted mean estimates of the models; orange vertical bars represent the 95% CrI. Grey dots represent raw data, horizontally jittered. Numbers represent the posterior probability of a mean difference between compared estimates. A statistically meaningful effect (presented in bold) can be assumed when the posterior probability of the mean difference between compared estimates is higher than 0.95.

Diagonal reaction distance (rz) varied among types of social units. Family groups, female groups and mixed groups had higher rz, compared with solitary males, while bachelors rz was comparable to the reference level (Table 3, Fig. 4). The reaction thresholds estimated from the model parameters represent the minimum UAS heights at which it is possible to fly over each social unit without causing a flight reaction (Table 3).

**Table 3:** Results from a generalized linear model for diagonal reaction distance (rz) and estimated reaction thresholds. We present estimates of the parameters with their 95% credible intervals (CrI) in brackets. A statistically meaningful effect of a fixed factor (presented in bold) can be assumed if zero is not included within the 95% CrI or if the mean difference between compared estimates is higher than 0.95.

Model results: Diagonal reaction distance (rz)		Reaction thresholds	
Predictors	Mean estimate (95%CrI)	Type of social units	Predicted estimates of minimum flight heights (m) (95% CrI)
Intercept	**5.04 (4.86; 5.211)**	**Solitary**	153.84 (128.61–183.71)
Social unit: Family groups^a^	**0.35 (0.08; 0.62)**	**Family**	217.49 (176.57–268.50)
Social unit: Female groups^a^	**0.42 (-0.02; 0.87)**	**Female**	234.51(156.47–356.30)
Social unit: Bachelors^a^	0.16 (-0.19; 0.51)	**Bachelors**	180.93 (133.47–243.62)
Social unit: Mixed groups^a^	**0.81 (0.52; 1.08)**	**Mixed**	344.34 (277.00–426.54)

^a^Reference level: Solitary males.

## Discussion

### Effect of sociability and group composition

We found a strong effect of sociability in the behavioural responses of wild guanacos to the drone’s approach. As expected, large groups were more reactive and less tolerant (i.e., greater flight distances) than small groups and solitary individuals. These results suggest that sociability allows guanacos to improve their capacity to react to human-associated disturbances, like drones, probably due to increased detection ability in larger groups. Previous studies showed both similar group-size effects for the same guanaco population^29,39^ and null^40,41^ or opposite effects in other populations^42^, depending on the type and disturbance history of the populations. This demonstrates the plasticity of the guanaco's social behaviour and is consistent with the intra-specific variability in the group size effects on flightiness observed in other ungulate species^22^.

Apart from group size, we cannot rule out other effects related to sociability that may be operating simultaneously. For example, the territoriality of family and solitary groups associated with the guanaco’s mating system^34^ could weigh on the decision to escape because these groups have costs associated with fleeing that the others do not^43^. Also, males and females tend to differ in risk perception^44^, and group types vary in sex ratio^45^. These effects may have less influence on the assessment of escape costs at the end of the reproductive season, which is when this study was conducted, than during the peak of the reproductive season when, for example, territorial males that flee are highly exposed to loss of territories and females. However, in order to conclude on the relative weight of group size and social unit type, specific studies evaluating escape responses in different social groups with comparable group size ranges would be necessary.

To our knowledge, this is the first study whose main purpose was to explicitly assess the effect of sociability on the probability and intensity of the behavioural responses to drone-induced disturbance and that reports a strong and conspicuous effect. Previous information for different species about this topic is scarce, inconclusive or even anecdotal. In some cases, group size was included simply as a potentially important factor among others, without an ecological theoretical framework. Vas et al.^12^ were the first authors to suggest that group size could influence the reaction distances to drones, observing more than 50 flamingos (*Phoenicopterus roseus*) reacting at maximum distances (25–30 m), although the authors recognized that their sample size was very low to confirm this trend. A review by Mulero-Pázmány et al.^15^ found that, for acrobatic or irregular drone flights, the animals (mostly birds) flew longer distances when in large groups, although a recent study reported that the effect of group size for 22 bird species was negligible^11^. Particularly, in terrestrial mammals, Bennitt et al.^16^ found no effect of group size on the probability of alertness and reaction in 7 species of African ungulates in response to drone approach. The social organization and the stability of grouping patterns of animal species can be complex and vary depending on reproductive strategies (e.g., territorial vs non-territorial), predator hunting strategies (e.g., stalking vs cursorial) and the stage during the life cycle (e.g., reproductive vs non-reproductive)^46–48^. Moreover, local ecological conditions and predation risk levels modulate social organization and group size between populations of the same species, including the guanaco^46,49^. In this sense, it is expected that gregarious species with different levels of stability in social organization, group cohesion and function as an anti-predatory strategy will respond differently to drone-induced disturbance. We recommend that studies assessing the potential impacts of drone use for wildlife or recreation should consider differences in sociability when designing and analysing their data.

Contrary to our expectations, the response of guanacos to the drone's approach was independent of the presence of offspring in the groups. Although we observed that the social units that showed greater reaction distances were those with offspring (family, female and mixed groups; Fig. 4), partial and indirect analyses showed that this response could be explained by differences in group size and not by the presence of offspring (Supplementary Note). In other words, groups with and without offspring reacted more frequently and at greater distances when they were larger (Figs. 2a, 3a). In general, higher fleeing responses from groups with offspring are common in ungulates^22^ and have also been reported in previous studies in guanacos exposed to different human-induced terrestrial disturbances, such as tourism^42^ or traffic^41,42^. The absence of this effect in our study could result from two factors acting separately or interactively. First, flight represents an energy expenditure and a potential short-term habitat shift that can be very costly at certain times of the year^50^. Our study was carried out at the end of the reproductive season, when offspring may be especially susceptible to the loss of reserves that allow them to face the first cold of the southern autumn and the migration process in search of better food resources. Second, the offspring’s vulnerability to predation is lower at this time than in the early post-natal period, when most of the annual offspring mortality occurs (i.e., 60%)^51^. Taken together, it is possible that at the time of the study, the mothers’ evaluate not anticipating the reaction response as they would do with better conditions of food availability and higher predation risk of the offspring. These explanations remain speculative and require more standardized experiments that, for example, compare different seasonal periods and use a more informative variable such as the number of offspring.

### Effect of flight plan characteristics

Not surprisingly, lower flight heights increased the probability of reaction. Similar results were reported in other terrestrial mammals^16,32^ but also in birds^11,52^, marine mammals^13,53^ and reptiles^21^, revealing that drones flying at low heights can be a threatening stimulus for a wide range of taxa. By contrast, the effect of drone speed is less clear. Contrary to our preliminary analysis^36^, we found no significant relationship between drone speed and the probability of behavioural reaction when other strong-effect variables—like group size—were included in the analysis. However, the animals that reacted to the drone's approach did so at a greater horizontal flight distance (rd) when the drone speed was lower, irrespective of the flight height. This result could arise from the relationship between the drone speed and the individual reaction time. With a similar average launch distance for both speeds (d0_*fast speed*_ = 525 ± 131 m, d0_*slow speed*_ = 526 ± 123 m), the mean reaction time of the guanacos was higher at slow speed (fast speed = 53.8 ± 27.8 s; slow seed = 97.2 ± 53.2 s). This data suggests that the probability of fleeing per time unit is not constant but increases with the decrease in the drone-guanacos distance; in that case, mean reaction time along with mean reaction distance should decrease non-linearly with speed. More than two measures of speed and reaction times are needed to explore this hypothesis and better understand the biological importance of drone approach speed as a disturbance factor.

### Reaction thresholds and minimum flight heights

Diagonal distance (rz) allowed us to identify the threshold flight height that should be followed to minimise behavioural disturbance to guanacos. In our study, even solitary individuals have high reaction distances, showing that, overall, the responsiveness of guanacos to the drone is the most dramatic reported so far for a wild species^13,21,54^. Even more, the difference in reaction distance compared with other ungulates is strikingly high—in African ungulates, it does not exceed 100 m^16^.

Detectability of the drone primarily involves the sensory (visual and auditory) abilities of the target species, but these can be shaped by factors such as environmental noise and disturbance history. According to the hearing ability under standard conditions of temperature and humidity, the average distance at which an ungulate (i.e., white-tailed deer, *Odocoileus virginianus*) hears a multirotor drone is 228.3 m (SE ± 39.4^55^), very close to the reaction thresholds calculated in this study for all social units except from mixed groups (Table 3). Particularly, the noise level of a Phantom drone similar to the one used in this study is between 70 and 80 dB (Phantom 4)^56,57^. Our guanaco population lives in an open desert environment without obstructive vegetation, where noise levels of the drone engines may be comparatively more audible than, for example, in coastal and shallow marine environments facing wind and wave sounds^58,59^ or in a penguin colony^60^. Additionally, it is well documented that both hunting pressure and low contact with human presence generate relatively more pronounced responses than perceived non-lethal human disturbances (i.e. tourism, traffic), and this is also true for populations of the same species^22,40^. The guanaco population in our study is subject to low levels of human disturbance, with little human presence and low poaching pressure. Overall, the high sensitivity to the drone documented here for guanacos may be the maximum expected for an ungulate when other factors such as environmental noise and disturbance history do not act as attenuators.

### Implications for research and monitoring of sensitive wildlife

**Figure 5:**
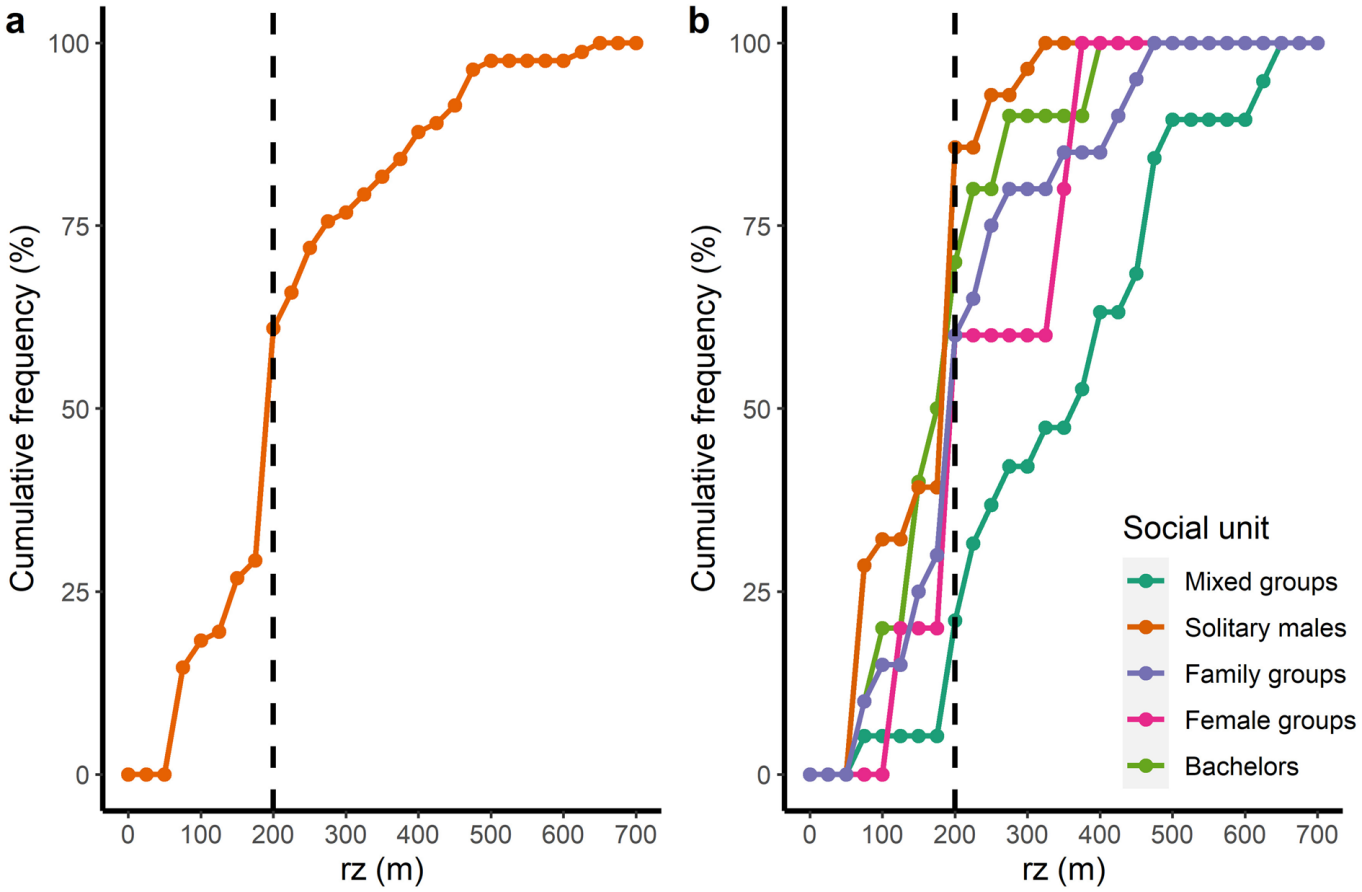
Cumulative frequency of rz for (**a**) all flights, and (**b**) each social unit separately. The dotted vertical lines at 200 m represent a trade-off between flying high enough to minimise flight reactions in the guanaco population under study, while ensuring safe flights and unbiased data, using a Phantom 4 Advanced (a multirotor drone carrying a 20MP sensor).

When drones are used for wildlife monitoring, disturbance to target species must be kept to a minimum to reduce behavioural responses that could lead to detection bias. If animals hide or flee before the UAS sensors can capture them, fewer individuals will be observed than are actually present, which could generate erroneous results^55^. At the same time, other factors such as sensor resolution (which decreases with flight height), flight safety and aviation authority regulations may be limiting. For highly reactive species or populations, there is a trade-off between flying high enough to prevent evasive behaviour while ensuring safe flights and unbiased data. In our experience, flying at 200 m AGL with a multirotor drone carrying a 20 MP sensor ensures minimising behavioural reaction, avoiding strong winds that can jeopardize the safety of equipment and operators, staying within height ranges allowed by the aeronautical authority with special permits^61,62^ and detecting adult guanacos without counting errors^36^. However, there is still much to improve. Plotting the cumulative frequency of rz shows that at this height, approximately 40% of the guanacos (Fig. 5a), mostly from mixed groups (Fig. 5b), would react before the drone flies overhead, probably underestimating the counts. To further reduce reaction distances of sensitives species, we recommend that researchers: (1) employ quieter, small multirotor drones with the same or better sensor performance; (2) use low-noise propellers; (3) when possible, conduct surveys when very large groups are less common in the population (e.g., the mating season) in cases when there is a strong effect of group size; and (4) assess if animals habituate to repeated drone exposure, as it was found in other taxa^63–65^.

Overall, our study provides new evidence to better understand the complexity of species' behavioural responses to a novel human-induced disturbance, like drones. In particular, our results demonstrate the importance of considering sociability and natural history as factors shaping species’ reactions, not only to design accurate flight plans but also to develop the animal welfare protocols that should be associated with the use of drones, whether scientific or recreational.

## Methods

### Ethics declarations

The study was carried out under the permits RESOL-2019-154-E by the Natural Resources Department from the Government of Mendoza, and ANAC Nro 00005366 and IF-2019-68620267-APN-DNSO#ANAC from the National Civil Aviation Administration.

### Study site and fieldwork

This study was conducted in Northern Patagonia, in west-central Argentina during the late guanaco reproductive season^66^ (austral summer of 2018). The study area included the northern part of the 665,000 ha La Payunia Reserve (between 36°00′ and 36°36′ S, and 68°34′ and 69°23′ W), a semiarid volcanic landscape of shrub and grassland steppes^67^. Vegetation is xerophytic, with 58% plant cover, and corresponds to the La Payunia phytogeographic province^67^. The study area holds the largest population of *L. guanicoe* of the west-central region of Argentina, with about 26,000 individuals in spring in the northern part of the reserve^68^. This is one of only a few guanaco populations that maintain annual migrations^69^. The main predator of guanacos is the puma (*Puma concolor*) and at La Payunia, predation is the most frequent cause of death for guanacos^70^.

In this region, human activities are limited. Tourism activity and tourist access is restricted and controlled by park rangers (Aros L., personal communication); guanaco mortality due to poaching is low (< 1%;^70^); and few humans inhabit the study area. The dominant land use is livestock grazing, primarily by goats^71,72^.

### Flight plans and behavioural records

We used a Phantom 4 Advanced (DJI, Shenzhen, China), a small quadcopter with an on-board 20-megapixel camera (colour: white, Diagonal Size (propellers excluded): 350 mm, weight: 1368 g, max. speed (P-mode): 50 km h^-1^, max wind speed resistance: 36 km h^-1^). Further technical specifications can be found at https://www.dji.com/phantom-4-adv/info#specs.

We performed horizontal flights as described in^36^. Briefly, the drone was launched at a minimum distance of 300 m from each guanaco group and flew towards it at constant height and speed (Fig. 1). We combined low (2–4 m s^−1^) and fast (8–10 m s^−1^) speeds with low and high heights (60 and 180 m AGL, respectively), resulting in 4 combinations of speed and height treatments. One operator steered the drone, and a second person observed the guanaco groups’ behaviour from the ground using binoculars (10 × 42 mm; Vanguard) and a 60-mm spotting scope (20–60 × ; Bushnell Trophy XLT) and recorded the changes in behaviour using a digital recorder (Panasonic RR-US551). We considered all the animals as a focal group^73^ and recorded whether there was a flight reaction of the animals before the drone was positioned above the group (Fig. 1). We classified guanaco responses as (i) flight reaction (walking quickly or running away from the guanaco’s original location in the opposite direction to the UAS), or (ii) no reaction, that included apparent lack of detection (the animals continued displaying the same behaviour recorded before the UAS flight) or UAS detection (alert posture, with the animal standing with its head and neck upright, ears erect and aimed directly at the stimulus)^29^. We considered a flight reaction to have occurred for the group when at least one of the individuals of the focal group exhibited a fleeing response, followed by the others^36^. All flights were conducted in open habitats with low grasslands/shrublands to avoid variability in animal’s risk perception^29,74^. For every flight, we recorded and classified the time as morning (7:00–11:00), midday (11:00–15:00) or afternoon (15:00–20:00), and measured average and maximum wind speed using an anemometer (Trotec BA06). At wind speeds higher than 7 m s^-1^ we decided not to fly for the safety of the operator and the aircraft.

When animals were visibly disturbed by our presence, flights and observations were not initiated. The observer recorded the type of social unit and group size in the field by counting the number of adults and offspring, based on body size. Groups were identified by excluding, at the beginning of each observation, individuals more than 300 m away from their neighbours. In each case, this was confirmed by the movement of the animals during the observations (i.e., the members of the same group moved together in the same direction, while the other individual(s) stayed in the same place or moved in another direction)^29,38^. As there is no obvious sexual dimorphism, guanaco sex was determined only after observing the testes^34^.

### Distance estimations

We estimated horizontal reaction distances (rd, Fig. 1) in the field, defined as the distance between the guanaco group and the drone's projection to the ground at the moment of reaction, as follows. At the beginning of each flight, we identified a guanaco group and registered spatial cues (e.g., shrubs, hills) that helped us to determine its exact original position. First, we measured the approximate distance between the group and the take-off point (d0) with a laser rangefinder (Leica LRF 800), which helped us determine how far the drone should fly. Then, the drone pilot ascended the UAS vertically to the desired height and approached the group in a straight line until it reached the group’s original location. For each flight, we obtained videos (Supplementary Video) using the DJI GO software, which contains a video caption option that allowed us to record the telemetry information (speed, flight time and advance distance of the drone) on the screen during the entire flight. The drone's advance was synchronized with the start of the behavioural observations. The observer recorded the changes in behaviour, as described above. At the end of each approach, we confirmed the distance between the take-off point of the drone and the group´s original location (d0) using a hand-held Global Positioning System (Garmin eTrex 10). To do this, we marked a waypoint at the take-off point, walked in a straight line to the group’s original position and used the tool “Go to”, which displayed the distance between the two points. Finally, we synchronized the drone's video with the behavioural records to estimate how much the drone advanced until the group of guanacos reacted (dx) and estimated rd using Eq. (2)

2 $$rd=d0-dx.$$

We estimated diagonal reaction distances (rz, Fig. 1), defined as the straight line distance from the guanaco group to the drone at the moment of reaction, following the trigonometric relationship in Eq. (3)

3 $$rz=\sqrt{{y}^{2}+{rd}^{2}}.$$

### Data analysis

To evaluate how social aggregation and offspring presence, in combination with flight plan characteristics (height and speed), influenced the behavioural response of wild guanacos to the drone's approach, we selected a sequential analysis that allowed us to integrate information from all flights^15^. First, we examined which explanatory variables determined the probability of a behavioural reaction (n = 90) by fitting generalized linear models (GLM^75^) with a binomial error distribution and a logit link function. Next, we focused only on the flights in which we observed behavioural reactions (n = 61) and analysed distance rd, by fitting GLM with a gamma error distribution and log link function. For both models, we first performed preliminary analyses assessing the effect of time of day and wind speed on the behavioural reaction of guanacos and found no significant effects, so we excluded them from the rest of the analysis. We considered group size (range = 1–159), drone height (60/80 m AGL), speed (2–4 m s^−1^/8–10 m s^−1^) and the presence of offspring (1 = presence, 0 = absence) as explanatory variables. We also considered the interaction terms group size*height and speed*height to assess whether the responses of group size or speed, respectively, varied with height. Group size was log-transformed to account for the few samples of very large groups of guanacos. Type of social unit has aliasing issues (i.e., linear dependency between covariates^75^) with group size and presence of offspring, which prevents the inclusion of these variables together in the same model. Thus, we fitted a new set of models similar to the previous ones but including the type of social unit instead of group size.

We considered rz (Fig. 1) as a reaction threshold since it is the minimum diagonal distance at which guanacos can be approached without causing a behavioural reaction, and at the same time, it represents the minimum height at which the drone can fly over the animals, minimising visual and/or auditory disturbance. We modelled rz (n = 82) using a GML with a gamma error distribution and log link function. We considered the type of social unit (solitary, family group, bachelors, females, mixed) as an explanatory variable. We excluded 8 flights from the analyses because we could not identify the type of social unit.

We tested the multicollinearity among predictors for each model by calculating the generalized variance inflation factor (GVIF)^76^, which is a generalization of the variance inflation factor (VIF). GVIF is applied to measure the collinearity among covariates, such as dummy regressors from a categorical variable. Fox and Monette^76^ suggest reporting the adjusted generalized variance inflation factors as shown in Eq. (4)

4 $$AGVIF={GVIF}^{1/(2df)}$$

where *df* is the number of degrees of freedom associated with the term and is analogous to reporting the square root of the VIF for a single coefficient. As a rule of thumb, AGVIF values greater than 2.236 (analogous to VIF > 5) were considered an indication of collinearity^77^. For the binomial and the rd models with group size, we excluded the interaction term group size*height because VIF values were slightly > 5 (6.06 and 5.17, respectively).

We used a Bayesian framework with non-informative priors to obtain 95% credible intervals (CrI) around the mean, representing the uncertainty around our estimates. To obtain the posterior distribution we simulated 10,000 values from the joint posterior distribution of the model parameters^78^. In all figures, we display raw data, the predicted estimate from the models and 2.5–97.5% CrI. We considered an effect to be statistically meaningful when the posterior probability of the mean difference between compared estimates was higher than 0.95 or when the estimated CrI did not include zero (for further details on statistical inference, see^79^). We also analysed model residuals using graphical methods (i.e. qqplots of residuals fitted values versus residuals) for homogeneity of variance or other departures from model assumptions and model fit. All modelling was performed with the software R v3.4.3^80^, using the packages “car”^81^, “stats”^80^ and “arm”^82^.

## Supplementary Information

Supplementary Information 1.

Supplementary Information 2.

## Data Availability

The datasets generated during and/or analysed during the current study are available from the corresponding author on reasonable request.

## References

[1] Jones GP, Pearlstine LG, Percival HF (2006). An assessment of small unmanned aerial vehicles for wildlife research. Wildl. Soc. Bull..

[2] Jones, G. P. The feasibility of using small unmanned aerial vehicles for wildlife research. Masters Thesis. (University of Florida, 2003).

[3] Watts AC (2008). Unmanned aircraft systems (UASs) for ecological research and natural-resource monitoring (Florida). Ecol. Restor..

[4] Chabot, D. Systematic Evaluation of a Stock Unmanned Aerial Vehicle (UAV) System for Small-Scale Wildlife Survey Applications. Masters Thesis. (McGill University, 2009).

[5] Koski WR (2009). Evaluation of an unmanned airborne system for monitoring marine mammals. Aquat. Mamm..

[6] Soriano P, Caballero F, Ollero A (2009). RF-based particle filter localization for wildlife tracking by using an UAV. Int. Symp. Robot..

[7] Sukkarieh, S. UAV based search for a radio tagged animal using particle filters at Stuttgart. In *Australasian Conference on Robotics and Automation (ACRA)* (2009).

[8] Abd-Elrahman A, Pearlstine L, Percival F (2005). Development of pattern recognition algorithm for automatic bird detection from unmanned aerial vehicle imagery. Surv. L. Inf. Sci..

[9] Singh KK, Frazier AE, Frazier AE (2018). A meta-analysis and review of unmanned aircraft system (UAS) imagery for terrestrial applications. Int. J. Remote Sens..

[10] Rebolo-Ifran N, Grilli MG, Lambertucci S (2019). Drones as a threat to wildlife: YouTube complements science in providing evidence about their effect. Environ. Conserv..

[11] Weston MA, O’Brien C, Kostoglou KN, Symonds MREE (2020). Escape responses of terrestrial and aquatic birds to drones: Towards a code of practice to minimize disturbance. J. Appl. Ecol..

[12] Vas E, Lescroël A, Duriez O, Boguszewski G, Grémillet D (2015). Approaching birds with drones: First experiments and ethical guidelines. Biol. Lett..

[13] Pomeroy P, O’Connor L, Davies P (2015). Assessing use of and reaction to unmanned aerial systems in gray and harbor seals during breeding and molt in the UK. J. Unmanned Veh. Syst..

[14] Giles AB (2020). Responses of bottlenose dolphins (Tursiops spp.) to small drones. Aquat. Conserv. Mar. Freshw. Ecosyst..

[15] Mulero-Pázmány M (2017). Unmanned aircraft systems as a new source of disturbance for wildlife: A systematic review. PLoS ONE.

[16] Bennitt E, Bartlam-Brooks HLA, Hubel TY, Wilson AM (2019). Terrestrial mammalian wildlife responses to unmanned aerial systems approaches. Sci. Rep..

[17] Irigoin-Lovera C, Luna DM, Acosta DA, Zavalaga CB (2019). Response of colonial Peruvian guano birds to flying UAVs: Effects and feasibility for implementing new population monitoring methods. PeerJ.

[18] McEvoy JF, Hall GP, McDonald PG (2016). Evaluation of unmanned aerial vehicle shape, flight path and camera type for waterfowl surveys: Disturbance effects and species recognition. PeerJ.

[19] Barnas AF, Felege CJ, Rockwell RF, Ellis-Felege SN (2018). A pilot(less) study on the use of an unmanned aircraft system for studying polar bears (*Ursus maritimus*). Polar Biol..

[20] Jarrett D, Calladine J, Cotton A, Wilson MW, Humphreys E (2020). Behavioural responses of non-breeding waterbirds to drone approach are associated with flock size and habitat. Bird Study.

[21] Bevan E (2018). Measuring behavioral responses of sea turtles, saltwater crocodiles, and crested terns to drone disturbance to define ethical operating thresholds. PLoS One.

[22] Stankowich T (2008). Ungulate flight responses to human disturbance: A review and meta-analysis. Biol. Conserv..

[23] Weston MA, Mcleod EM, Blumstein DT, Guay PJ (2012). A review of flight-initiation distances and their application to managing disturbance to Australian birds. Emu.

[24] Wisdom, M. J., Ager, A. A., Preisler, H. K., Cimon, N. J. & Johnson, B. K. Effects of off-road recreation on mule deer and elk. In *Transactions of the 69th North American Wildlife and Natural Resources Conference* 531–550 (2004).

[25] Penny SG, White RL, Scott DM, MacTavish L, Pernetta AP (2019). Using drones and sirens to elicit avoidance behaviour in white rhinoceros as an anti-poaching tactic. Proc. R. Soc. B Biol. Sci..

[26] Frid A, Dill LM (2002). Human-caused disturbance stimuli as a form of predation risk. Conserv. Ecol..

[27] Dill LM, Frid A (2020). Behaviourally mediated biases in transect surveys: A predation risk sensitivity approach. Can. J. Zool..

[28] Pulliam RH (1973). On the advantage of flocking. J. Theor. Biol..

[29] Taraborelli P, Gregorio P, Moreno P, Novaro A, Carmanchahi P (2012). Cooperative vigilance: The guanaco’ s (*Lama guanicoe*) key antipredator mechanism. Behav. Process..

[30] Delm MM (1990). Vigilance for predators: Detection and dilution effects. Behav. Ecol. Sociobiol..

[31] Roberts G (1996). Why individual vigilance declines as group size increases. Anim. Behav..

[32] Brunton E, Bolin J, Leon J, Burnett S (2019). Fright or flight? Behavioural responses of kangaroos to drone-based monitoring. Drones.

[33] Lent PC (1974). Mother-infant relationships in ungulates. Behav. Ungulates Relat. Manag..

[34] Franklin W (1983). Contrasting socioecologies of South America´s wild camelids: The vicuña and the guanaco. Adv. Study Mamm. Behav..

[35] Ortega IM, Franklin WL (1995). Social organization, distribution and movements of a migratory guanaco population in the Chilean Patagonia. Rev. Chil. Hist. Nat..

[36] Schroeder NM, Panebianco A, Musso RG, Carmanchahi P (2020). An experimental approach to evaluate the potential of drones in terrestrial mammal research: A gregarious ungulate as a study model. R. Soc. Open Sci..

[37] Lima SL (1995). Back to the basics of anti-predatory vigilance: the group size effect. Anim. Behav..

[38] Marino A, Baldi R (2008). Vigilance patterns of territorial guanacos (*Lama guanicoe*): The role of reproductive interests and predation risk. Ethology.

[39] Taraborelli P (2014). Different factors that modify anti-predator behaviour in guanacos (Lama guanicoe). Acta Theriol. (Warsz).

[40] Donadio E, Buskirk SW (2006). Flight behavior in guanacos and vicuñas in areas with and without poaching in western Argentina. Biol. Conserv..

[41] Marino A, Johnson A (2012). Behavioural response of free-ranging guanacos (*Lama guanicoe*) to land-use change: Habituation to motorised vehicles in a recently created reserve. Wildl. Res..

[42] Malo JE, Acebes P, Traba J (2011). Measuring ungulate tolerance to human with flight distance: A reliable visitor management tool?. Biodivers. Conserv..

[43] Marino A (2012). Indirect measures of reproductive effort in a resource-defense polygynous ungulate: Territorial defense by male guanacos. J. Ethol..

[44] Andrea M, Ricardo B (2008). Vigilance patterns of territorial guanacos (*Lama guanicoe*): the role of reproductive interests and predation risk. Ethology.

[45] Merino ML, Cajal CJ (1993). Estructura social de la población de guanacos (*Lama guanicoe* Muller, 1776) en la costa norte de Península Mitre, Tierra del Fuego, Argentina. Stud. Neotrop. Fauna Environ..

[46] Marino A, Baldi R (2014). Ecological correlates of group-size variation in a resource-defense ungulate, the sedentary Guanaco. PLoS ONE.

[47] Fattorini N (2019). Temporal variation in foraging activity and grouping patterns in a mountain-dwelling herbivore: Environmental and endogenous drivers. Behav. Process..

[48] Blank D, Ruckstuhl K, Yang W (2012). Influence of population density on group sizes in goitered gazelle (*Gazella subgutturosa* Guld., 1780). Eur. J. Wildl. Res..

[49] Isvaran K (2007). Intraspecific variation in group size in the blackbuck antelope: The roles of habitat structure and forage at different spatial scales. Oecologia.

[50] Mahoney SP, Mawhinney K, McCarthy C, Anions D, Taylor S (2001). Caribou reactions to provocation by snowmachines in Newfoundland. Rangifer.

[51] Ruiz Blanco, M. *et al.* Supervivencia y causas de mortalidad durante el primer año de vida de guanacos en el norte de patagonia. In *XXVII Jornadas Argentinas de Mastozoología* 151 (2014).

[52] Weimerskirch H, Prudor A, Schull Q (2018). Flights of drones over sub-Antarctic seabirds show species- and status-specific behavioural and physiological responses. Polar Biol..

[53] McIntosh, R. R., Holmberg, R. & Dann, P. Looking without landing-using Remote Piloted Aircraft to monitor fur seal populations without disturbance. *Front. Mar. Sci.* **5**, (2018).

[54] Mesquita GP, Rodríguez-Teijeiro JD, Wich SA, Mulero-Pázmány M (2020). Measuring disturbance at swift breeding colonies due to the visual aspects of a drone: a quasi-experiment study. Curr. Zool..

[55] Scobie CA, Hugenholtz CH (2016). Wildlife monitoring with unmanned aerial vehicles: Quantifying distance to auditory detection. Wildl. Soc. Bull..

[56] Rümmler MC, Esefeld J, Hallabrin MT, Pfeifer C, Mustafa O (2021). Emperor penguin reactions to UAVs: First observations and comparisons with effects of human approach. Remote Sens. Appl. Soc. Environ..

[57] Zbyryt A, Dylewski Ł, Morelli F, Sparks TH, Tryjanowski P (2020). Behavioural responses of adult and young White Storks Ciconia ciconia in nests to an unmanned aerial vehicle. Acta Ornithol..

[58] Christiansen F, Rojano-Doñate L, Madsen PT, Bejder L (2016). Noise levels of multi-rotor unmanned aerial vehicles with implications for potential underwater impacts on marine mammals. Front. Mar. Sci..

[59] Arona L, Dale J, Heaslip SG, Hammill MO, Johnston DW (2018). Assessing the disturbance potential of small unoccupied aircraft systems (UAS) on gray seals (*Halichoerus grypus*) at breeding colonies in Nova Scotia, Canada. PeerJ.

[60] Goebel ME (2015). A small unmanned aerial system for estimating abundance and size of Antarctic predators. Polar Biol..

[61] Cracknell AP (2017). UAVs: Regulations and law enforcement. Int. J. Remote Sens..

[62] ANAC, A. N. de A. C. *Reglamento de Vehículos Aéreos no Tripulados (VANT) y de Sistemas de Vehículos Aéreos no Tripulados (SVANT)*. (2019).

[63] Brisson-Curadeau É (2017). Seabird species vary in behavioural response to drone census. Sci. Rep..

[64] Rümmler M-C, Mustafa O, Maercker J, Peter H-U, Esefeld J (2018). Sensitivity of Adélie and Gentoo penguins to various flight activities of a micro UAV. Polar Biol..

[65] Ditmer MA (2019). Bears habituate to the repeated exposure of a novel stimulus, unmanned aircraft systems. Conserv. Physiol..

[66] Young JK, Franklin WL (2004). Territorial Fidelity of male guanacos in the Patagonia of Southern Chile. J. Mammal..

[67] Martínez Carretero E (2004). La Provincia Fitogeográfica de la Payunia. Boletín la Soc. Argentina Botánica.

[68] Schroeder NM (2014). Spatial and seasonal dynamic of abundance and distribution of guanaco and livestock: Insights from using density surface and null models. PLoS One.

[69] Bolgeri, M. J. Caracterización de movimientos migratorios en guanacos (*Lama guanicoe*) y patrones de depredación por pumas (*Puma concolor*) en la Payunia, Mendoza. Phd Thesis. (Universidad Nacional del Comahue, 2016).

[70] Bolgeri MJ, Novaro AJ (2015). Variación espacial en la depredación por puma (*Puma concolor*) sobre guanacos (*Lama guanicoe*) en la Payunia, Mendoza,Argentina. Mastozoología Neotrop..

[71] Candia R, Puig S, Dalmasso A, Videla F, Martínez Carretero E (1993). Diseño del Plan de Manejo para la reserva provincial La Payunia (Malargüe, Mendoza). Multequina.

[72] Carmanchahi PD (2011). Physiological response of wild guanacos to capture for live shearing. Wildl. Res..

[73] Martin, P. & Bateson, P. *Measuring Behaviour. An Introductory Guide*. (Cambridge University Press, 2007).

[74] Ydenberg RC, Dill LM (1986). The economics of fleeing from predators. Adv. Study Behav..

[75] McCullagh, P. & Nelder, J. *Generalized Linear Models. Second Edition*. (Chapman & Hall, 1989).

[76] Fox J, Monette G (1992). Generalized collinearity diagnostics. J. Am. Stat. Assoc..

[77] Zuur, A. F., Ieno, E. N. & Smith, G. M. *Analysing Ecological Data*. (Springer, 2007).

[78] Gelman, A. & Hill, J. Data analysis using regression and multilevel/hierarchical models. Cambridge 651 (2007). 10.2277/0521867061

[79] Korner-Nievergelt, F. *et al. Bayesian Data Analysis in Ecology Using Linear Models with R, BUGS, and Stan*. (2015). 10.1007/s13398-014-0173-7.2

[80] R Core Team. R Development Core Team. *R A Lang. Environ. Stat. Comput.* 55, 275–286 (2016).

[81] Fox, J. & Weisberg, S. *An R Companion to Applied Regression*, 3rd edn. (2019).

[82] Gelman A, Su Y, Yajima M, Hill J, Pittau MG, Zheng, Kerman J, Zheng T, Dorie V (2018). Generalized collinearity diagnostics. R package version.

