# Supplementary material for: Sociability strongly affects the behavioural responses of wild guanacos to drones
Source: Sci Rep. 2021 Oct 22;11:20901. doi: 10.1038/s41598-021-00234-5 (PMC8536753; doi:10.1038/s41598-021-00234-5)
Supplement: Supplementary file 1 — Supplementary Information 1. [file 41598_2021_234_MOESM1_ESM.pdf]

Sociability strongly affects the behavioural responses of wild guanacos to drones

Natalia M. Schroeder[1, 2, 3*] and Antonella Panebianco[2*]

[1]Instituto Argentino de Investigaciones de las Zonas Áridas, CONICET, CC 507, CP 5500 Mendoza, Argentina
[2]Grupo de Investigación en Eco-Fisiología de Fauna Silvestre (INIBIOMA-CONICET-AUSMA-UNCo), Pasaje de la paz 235, CP 8370 San Martín de los Andes, Neuquén, Argentina
[3]Facultad de Ciencias Agrarias, Universidad Nacional de Cuyo, Mendoza, Argentina

*Corresponding authors: Natalia M. Schroeder (natalias@mendoza-conicet.gob.ar); Antonella Panebianco (apanebianco@comahue-conicet.gob.ar)

Supplementary Note

Since it was not possible to incorporate both presence of offspring and type of social unit in the same model due to aliasing issues, we performed additional partial analyses to evaluate the effect of offspring presence on rz distances.

Using information in Fig. 4, we observed that groups with offspring (family, female, mixed) have larger rz distances than those without offspring (bachelors, solitary). When the presence of offspring was considered instead of type of social unit, there was an effect of this variable on rz (Table S1, Fig. S1a). Additionally, groups with offspring are the most numerous (Fig S1b).

Table S1. Results from a generalized linear model for diagonal reaction distance (rz) as a function of the presence of offspring. We present estimates of the parameters with their 95% credible intervals (CrI) in brackets. A statistically meaningful effect of a fixed factor (presented in bold) can be assumed if zero is not included within the 95% CrI or if the mean difference between compared estimates is higher than 0.95, and are presented in bold font.

**Predictors**	**Mean estimate (95%CrI)**
Incercept	**5.09 (4.94; 5.24)**
Presence of offspring: yes[a]	**0.54 (0.33; 0.75)**

[a]Reference level: without offspring

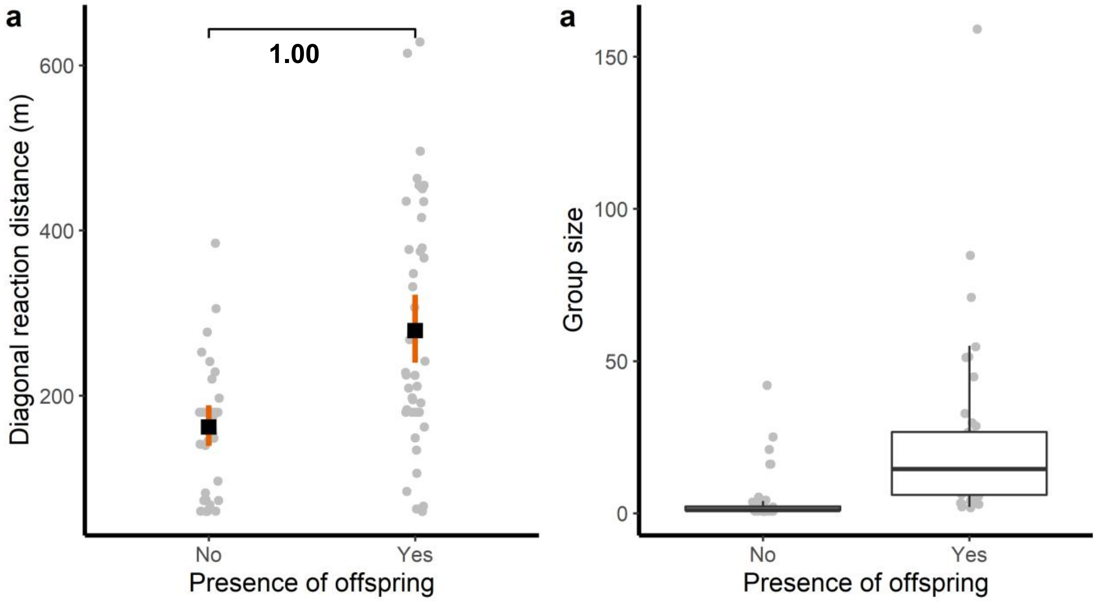

Figure S1. (a) Diagonal reaction distance (rz) of groups with and without offspring during experimental drone flights. Black squares represent mean estimates of the models; orange vertical bars represent the 95% CrI. Grey dots represent raw data, horizontally jittered. Numbers represent the posterior probability of a mean difference between compared estimates. A statistically meaningful effect (presented in bold) can be assumed when the posterior probability of the mean difference between compared estimates is higher than 0.95. **(b)** Boxplots for group size in groups with and without offspring.

In order to assess whether differences in rz distances between type of social units were due to the presence of offspring or to group size, we analyzed rz distances between family groups (with offspring) and bachelors (without offspring), which have variable group sizes but within a similar range of values. In this way, we excluded the potential effect of social units that have extreme group size values (solitary and mixed groups, Table 1). The results showed a positive effect of group size, but no difference of rz between family and bachelor groups (Table S2, Figure S2). This confirms that the increase in the flight response rz was explained by an increase in group size rather than by the presence of offspring.

Table S2. Results from a generalized linear model for diagonal reaction distance (rz), including only family groups and bachelors. We present estimates of the parameters with their 95% credible intervals (CrI) in brackets. A statistically meaningful effect of a fixed factor (presented in bold) can be assumed if zero is not included within the 95% CrI or if the mean difference between compared estimates is higher than 0.95, and are presented in bold font.

**Predictors**	**Mean estimate (95%CrI)**
Intercept	**4.62 (4.10; 5.15)**
Log(group size)	**0.27 (0.05; 0.49)**
Presence of offspring: yes[a]	0.24 (-0.12; 0.58)

[a]Reference level: without offspring

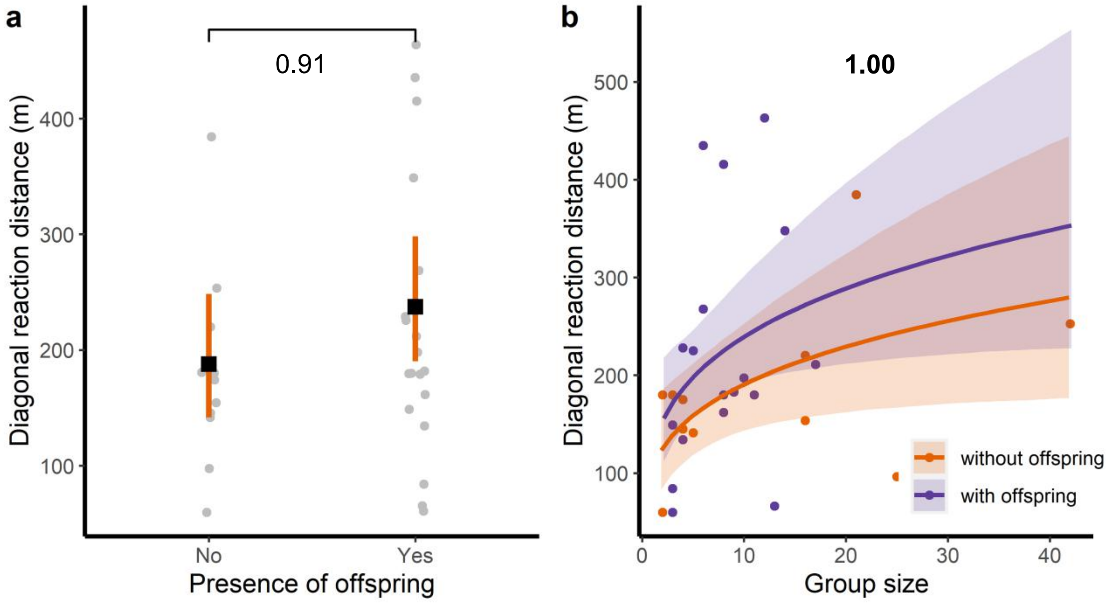

Figure S2. Relationship between diagonal reaction distance (rz), according to the presence of offspring (**a**), and group size (**b**), during experimental drone flights. Colour lines and black squares represent mean estimates of the models; colour bands and vertical bars represent the 95% CrI. Dots represent raw data, horizontally jittered in the case of (**a**). Numbers represent the posterior probability of a mean difference between compared estimates. A statistically meaningful effect can be assumed when the posterior probability of the mean difference between compared estimates is higher than 0.95.
