# Supplementary material for: Sociability strongly affects the behavioural responses of wild guanacos to drones
Source: Sci Rep. 2021 Oct 22;11:20901. doi: 10.1038/s41598-021-00234-5 (PMC8536753; doi:10.1038/s41598-021-00234-5)
Supplement: Supplementary file 2 — Supplementary Information 2. [file 41598_2021_234_MOESM2_ESM.pdf]

Supplementary Video

Available at the following link:

https://drive.google.com/file/d/1HijP2aLG7pYt2edEm1jLcIi4WJvZ-QDt/view?usp=sharing
